# The Perceived Role of Green Spaces on Mental Well‐Being in Adults Living in Regional Communities: A Qualitative Study

**DOI:** 10.1002/hpja.70072

**Published:** 2025-07-10

**Authors:** Hirbo Shore Roba, Stuart J. H. Biddle, Tracy Kolbe‐Alexander

**Affiliations:** ^1^ School of Health and Medical Sciences University of Southern Queensland Australia; ^2^ Centre for Health Research University of Southern Queensland Australia; ^3^ School of Public Health, College of Health and Medical Sciences Haramaya University Ethiopia; ^4^ Manna Institute, Centre for Health Research University of Southern Queensland Australia; ^5^ UCT Research Centre for Health Through Physical Activity, Lifestyle and Sport (HPALS), Division of Research Unit for Exercise Science and Sports Medicine University of Cape Town South Africa

**Keywords:** green space, mental health, mental well‐being, regional area, rural

## Abstract

**Issue Addressed:**

Research in Australia has shown that green spaces enhance mental health. However, most studies focused on urban areas, leaving regional settings underexplored. This study explores the perceived role and features of green spaces in supporting the mental health and well‐being of adults in regional Southeast Queensland, Australia.

**Methods:**

Semi‐structured interviews were conducted with 11 community residents aged 42–76 years from the Toowoomba Regional Council (TRC) area, and two focus group discussions (FGDs) were held with eight council officers. Transcripts were analysed thematically using NVivo.

**Results:**

Three key themes were identified regarding the role of green spaces: stress relief and restoration, spaces for physical and social activities, and barriers to and facilitators of green space use and activity engagement. Participants described green spaces as retreats from daily demands, fostering stress recovery, emotional balance and self‐reflection. Green spaces also supported physical activity and encouraged social interaction. Accessibility, perceived safety, maintenance and environmental conditions were identified as factors influencing usage and associated health benefits of green spaces.

**Conclusion:**

Green space in regional settings offers multifaceted benefits for mental health and well‐being, including restorative effects, opportunities for physical activity and social connection. However, accessibility, safety and seasonal challenges influence the use of green spaces and health outcomes.

**So What?:**

This study highlights the importance of green spaces for mental health in regional Australia, underscoring the need for context‐specific planning and management to optimise health benefits. These findings may inform policies and interventions to enhance green space quality, accessibility and utilisation in regional contexts.

## 1 Introduction

Greenspaces are natural or semi‐natural vegetated areas in public and private spaces, including parks, sports grounds, gardens and tree‐lined streetscapes [1]. Exposure to these spaces is associated with positive mental health outcomes [2, 3], psychological well‐being, and better quality of life [2, 4]. Conversely, environments with low vegetation cover and degraded landscapes are associated with adverse mental health outcomes [5].

Exposure to green space also promotes physical activity and social connections, both of which enhance mental well‐being [3, 6]. Physical activity undertaken within green spaces reduces stress [3, 5, 6], anxiety and depressive symptoms [7]. Additionally, green spaces promote social interactions, fostering social cohesion and a sense of community, thereby enhancing mental health and mental well‐being [3, 6]. Evidence also suggests that green spaces are associated with lower levels of loneliness [3, 8]. This underscores the importance of green spaces in promoting physical and social well‐being, thereby contributing to mental health.

In Australia, green spaces generally have a positive effect on mental health and well‐being outcomes [9, 10, 11, 12, 13, 14, 15]. However, these studies have predominantly focused on major cities, with limited attention paid to regional or rural settings [16, 17]. Focusing on cities might overlook the relationship between greenspaces and mental health outcomes in less urbanised settings, where the contexts differ. A systematic review by Browning et al. [18] highlighted that while half of the analyses showed comparable green spaces in cities and regional settings, 38% showed positive effects in cities and only 10% indicated more benefits in regional settings. These disparities suggest that greenspace research in cities may not be generalisable to rural settings, underscoring the need for research focusing on regional settings.

The framework proposed by Markevych et al. (6) was considered to advance our understanding of the role of greenspaces in mental health. This conceptual framework identifies three domains through which green space influences mental health: harm reduction, restoration and facilitation of health‐related behaviours [6]. Harm reduction includes mitigating environmental stressors such as noise, heat and air pollution [19]. Restoration, including stress recovery, is supported by Attention Restoration Theory (ART), which posits that natural environments replenish directed attention [19], and Stress Reduction Theory (SRT), which suggests nature exposure reduces psychophysiological stress [20]. Additionally, green space provides safe and accessible environments that promote physical activity and social cohesion [3, 6].

The socioecological model, influenced by Bronfenbrenner's ecological model of human development, provides a framework for examining the interaction between individual, environmental and social determinants of health [20]. This model elucidates how access to and usage of green spaces are influenced by personal factors, environment, greenspace characteristics and climatic conditions [21]. This framework has been used to explore personal and environmental factors associated with safety perceptions in urban green spaces [22] and to assess the association between green spaces and mental health [23].

Building on these frameworks, this study explores the perceived role and features of green spaces on mental health and well‐being in adults living in regional Southeast Queensland, Australia. Integrating theoretical frameworks provides a comprehensive understanding of the health effects of green spaces in regional settings and offers insights to guide public health and green space planning.

## 2 Methods

This qualitative study was conducted in the Toowoomba Regional Council (TRC) area, classified under the Modified Monash Model (MMM 2019), ranging from MM 2 to MM 5. MM 1 represents major cities, whereas MM 2–MM 7 are regional, rural or remote [24]. Located 127 km west of Brisbane, Toowoomba is the main urban centre of the TRC, with a population of 181 821 in 2023. The TRC manages 566 open spaces, covering 7771 ha [25].

### 2.1 Participants

Community residents aged 18 years and older living in the TRC area were invited to participate in interviews. The research team (H.S.R., S.J.H.B. and T.K.‐A.) contacted members of community groups, such as walking, hiking, bushwalking clubs, using existing networks and also via e‐mail, inviting them to participate in the study. Snow‐ball sampling was also used, whereby participants were encouraged to invite their peers to join in the study. In addition, this study was also publicised in the TRC's ‘ACTIVE’ newsletter. Local TRC officers involved in green space provision, planning and maintenance services from departments such as Park and Recreational Service, Transport and Drainage Planning, Conservation and Pest Management, and Natural Resources and Environment were invited to participate in focus group discussions (FGDs). Including officers in FGDs provided insights into the operation, management, provision and maintenance of green spaces, complementing perspectives of community participants. Participants received an information sheet detailing the purpose of the study and the data collection procedures. Consent was obtained from each participant before the interview or group session. Ethical approval was obtained from the Human Research Ethical Committee of the University of Southern Queensland (UniSQ) (reference number: ETH2023‐0575).

### 2.2 Data Collection

Semi‐structured interviews and FGDs were conducted either in person or online via Microsoft Teams, based on participants preferences. Author (H.S.R.), a trained interviewer and FGD facilitator, conducted interviews and moderated group discussions. He had no prior relationship with participants, lived in a metropolitan area with intermittent access and use of green space. A semi‐structured guide was used for both interviews and FGDs to address the research questions: ‘How do green spaces impact daily life and well‐being?’ and ‘which elements of green spaces have impact on promoting health and well‐being?’ Face‐to‐face interviews were recorded using a digital voice recorder, and online interviews and FGDs were recorded using Microsoft Teams. Face‐to‐face interviews were transcribed using the Dictate feature in Microsoft Word (Microsoft 365), and online interviews and FGDs were transcribed in real‐time using Microsoft Teams.

### 2.3 Data Analysis

All interview and FGDs transcripts were reviewed and corrected verbatim using audio recordings to ensure accuracy by the lead author (H.S.R.). Final transcripts were imported into NVivo 14 for thematic analysis, guided by Clarke and Braun's six‐step framework [26]. First, H.S.T. and T.K.A. reviewed the transcripts and familiarised themselves with the data. Then, H.S.R. generated initial codes and developed preliminary themes and subthemes using a deductive approach informed by Markevych et al. [6] theoretical framework constructs, including harm reduction, restoration and facilitation of health‐related behaviours, and the socioecological model, focusing on individual, environmental and social factors [21]. The codes, themes and subthemes were independently reviewed by T.K.‐A. H.S.R. and T.K.‐A. then discussed and agreed upon the coding structure, themes and subthemes. The research team subsequently reviewed and further refined themes and subthemes through iterative discussions to ensure trustworthiness and rigour. Analyses of the interview and FGDs transcripts were conducted separately, with overlapping and related themes integrated to form a cohesive thematic structure. Final themes and subthemes are supported by detailed descriptions, interpretations and illustrative participant quotes. In this study, ‘Community’ refers to a community resident, and ‘Officer’ refers to a TRC officer.

## 3 Results

Of the 18 community members who expressed an interest in participating, 11 interviews were conducted. Participants were from suburbs or localities of the Toowoomba Region, with six from MM 2 areas, four from MM 1 areas, and one from MM 5 area (small rural town). Their ages ranged from 41 to 74 years, and nine were female. The average duration of the interviews was 31 min, ranging from 18 to 48 min. Eight council officers from the Departments of Park and Recreational Service, Transport and Drainage Planning, Conservation and Pest Management, and Natural Resources and Environment attended one of two sessions. Their ages ranged from 30 to 55 years, and the majority were female (*n* = 5). The focus group sessions lasted for 40 and 55 min.

Three themes were identified after repeated reading of transcripts: stress relief and restoration, space for physical and social activities, and barriers and facilitators of green space use and activity engagement. The first two themes describe the perceived role of green spaces in promoting mental, physical and social well‐being, whereas the third theme highlights the contextual factors that influence the access to and usability of green spaces (Table 1).

**TABLE 1:** Thematic Analysis Framework.

Themes	Description	Subthemes	Quotes
Stress relief and restoration	This theme emphasises on the role of green space in offering an escape from everyday life, alleviating stress, fostering self‐reflection, thereby supporting emotional recovery	Escape	*“I think it is really important to have those green spaces because they take you away from all the tech, the rush and bustle.”* (Female, 56 years, Community) *“… it has a very wild, unkept space with a creek running through it. … you can go and sit there; you literally feel like you are not a city at all.”* (Female, 52 years, Community)
		Stress reduction and restoration	*“… just being in a space close to nature can actually start to calm the nervous system down.”* (Officer) *“It is nice to be in that environment and you come back home, and you feel much more ready to start the day much calmer about the day ahead instead of worrying about it from the moment you wake up”* (Female, 41 years, Community)
		Space for self‐reflection	*“For me being in those spaces, it takes me outside of my head into that space. So, I see things in a bigger perspective, a bigger picture, it is not just about me.”* (Female, 64, Community)
Space for physical activity and social connection	This theme highlights the role of green space as venues for promoting physical activity and fostering social interactions.	Physical activity	*“… you're more likely to go out for a walk or a run and engage in exercise if you're doing it in a nice space like a park, rather than just walking around suburban streets. … more motivating to engage in exercise… and does have direct health benefits.”* (Female, 41 years, Community). *“I tend to go to parks with my dogs and so being able take them with me and have them off‐leash, and throw balls for them… that is really important to me*” (Female, 52 years, Community)
		Social interaction and social connection	*“You see all walks of life, rich, poor, multicultural people, First Nations people… a whole mix of people together being friendly to each other.”* (Female, 65 years, Community) *“It is an opportunity to meet and greet other people as you are walking in parks. Sometimes, if you sit on benches … you are more likely to speak to somebody … because your mindset is outwards‐looking rather than inward‐looking. Your purpose for being in the park is to relax.”* (Female, 64 years, Community.) *“We live in a society where many a lot of people are lonely, living as a single person household. Connection is super important. Face‐to‐face communication with other people is a part of mental well‐being. So, if you don't know anybody in a local area … you can go to the park, meet somebody … that's the first person you've spoken to all day, it's a recognition and acknowledgement that you exist as a person.”* (Male, 76 years, Community).
Barriers and facilitators of green space use and activity engagement	This theme focusses on factors that either encourage or hinder green space usage and subsequently affecting health and well‐being benefits	Neighbourhood and green space characteristics	*“I think it's very accessible from people's homes and their walkability to those open space network.” (Officer)* *“It could also be about whether they can't find information about parks. So, I am thinking of how we have to make the web …. More accessible for people with vision disability”* (Officer)
		Safety	*“I would not go wandering by myself in Redwood Park. It is too isolated.”* (Female, 52 years, Community) *“… when it's still dark … you can still use the park, you stay on the footpath where you feel a bit safer because it's lit.”* (Female, 41 years, Community) *“I go out to Nelly Robinson Park if I want to go to a park but not my place because we have got homeless people there. So, you don't want to put yourself in that situation, so I avoid it.”* (Female, 69 years, Community) *“There is one area which has graffiti that I don't like walking along because it is dirty… they even tried to get rid of it, but they just leave. It looks worse, so I don't like that area.”* (Female, 64 years, Community)
		Seasonality	*“It was too dangerous; I had eight brown snakes around my house last year. Just coming out of nowhere. So, you need to be careful.”* (Male, 76 years, Community)

*Notes:* Community: community participants, Officer: Toowoomba Regional Council Officer.

## 4 Stress Relief and Restoration

### 4.1 Escape

Participants described green spaces as essential for escaping routine environments and meeting the demands of daily life. These spaces contrasted with everyday settings, providing relief from work, home and office confinement. The participants highlighted soundscapes, such as birdsong, rustling leaves and flowing water, as well as visual elements, such as trees, wildlife and open landscapes. These elements provided physical and psychological detachment, described as ‘stepping away’ to take fresh air or connect with nature:

I think it is really important to have those green spaces because they take you away from all the tech, the rush and bustle. (Female, 56 years, Community)

Some participants reported experiencing a sense of physical separation from urban settings in green spaces, describing the experience as ‘*not in the city*’ when surrounded by dense vegetation:

… it has a very wild, unkept space with a creek running through it. … you can go and sit there; you literally feel like you are not a city at all. (Female, 52 years, Community)

### 4.2 Stress Reduction and Restoration

Participants described that spending time in natural environments reduced stress, promoted calmness and restored emotions. They noted that spending time in tranquil and aesthetically appealing environments helps to relax, calm and alleviate tension. Watching wildlife, such as birds and lizards, along with natural soundscapes, was also noted to relieve stress and provide feelings of peace and emotional balance:

… just being in a space close to nature can actually start to calm the nervous system down. (Officer)

Participants noted that spending time in green spaces offers stability and grounding that centres thoughts and emotions. Natural features such as large trees, bushes, gardens and water play a key role in fostering grounding, maintaining mental balance and supporting a positive outlook:

It is nice to be in that environment and you come back home, and you feel much more ready to start the day much calmer about the day ahead instead of worrying about it from the moment you wake up (Female, 41 years, Community)

### 4.3 Space for Self‐Reflection

Participants described green spaces as environments that enhance self‐reflection and offer solitude and tranquil settings for introspection. These spaces allowed participants to shift their focus inward, contemplate personal and broader matters, and gain fresh insights into events or priorities that are most significant in life. One participant shared that nature journaling deepened their interactions with nature, encouraged mindfulness and focused on natural patterns:

For me being in those spaces, it takes me outside of my head into that space. So, I see things in a bigger perspective, a bigger picture, it is not just about me. (Female, 64, Community)

## 5 Space for Physical Activity and Social Connection

### 5.1 Physical Activity

Most participants noted that they frequently used green spaces to walk, which improved their physical fitness and provided health benefits. Diverse walking trails offer flexibility for short or extended walks, and options between concrete footpaths and natural trails or paved pathways. These features allowed the participants to tailor their physical activity to their fitness goals and preferences. Participants also noted that green spaces offer more motivating settings for physical activity than suburban streets or indoor environments:

… you're more likely to go out for a walk or a run and engage in exercise if you're doing it in a nice space like a park, rather than just walking around suburban streets. … more motivating to engage in exercise… and does have direct health benefits. (Female, 41years, Community).

Dog ownership has emerged as a key motivator of physical activity. For participants, walking dogs not only met the exercise needs of pets, but also encouraged them and their families to maintain an active lifestyle. Some noted that green spaces with off‐leash areas allowed interactive play with pets involving walking or running:

I tend to go to parks with my dogs and so being able take them with me and have them off‐leash and throw balls for them… that is really important to me. (Female, 52 years, Community)

### 5.2 Social Interaction and Social Connection

Participants noted that green spaces encourage casual and spontaneous social interactions perceived as less likely elsewhere. They emphasised that green spaces offer accessible areas for shared activities like dog walking, ‘Parkrun’, group walking, hiking, sitting and gardening. These activities create a relaxed atmosphere, facilitating conversations and connections, enhancing mental and social well‐being. Green spaces can also serve as community hubs, hosting events and multicultural festivals that draw people together, thereby creating a sense of unity and shared experiences:

You see all walks of life, rich, poor, multicultural people, First Nations people… a whole mix of people together being friendly to each other. (Female, 65 years, Community)

When elaborating on how green spaces foster casual and spontaneous interactions compared to urban streets, one participant noted:

It is an opportunity to meet and greet other people as you are walking in parks. Sometimes, if you sit on benches … you are more likely to speak to somebody … because your mindset is outwards‐looking rather than inward‐looking. Your purpose for being in the park is to relax. (Female, 64 years, Community.)

Participants noted that green spaces helped mitigate loneliness and social isolation. They highlighted that face‐to‐face communication or brief encounters in green spaces provide recognition, helping mitigate loneliness despite living alone. Participants expressed empathy for those experiencing loneliness due to illness or disability, and recognised community support in helping them access green spaces for nature and social interactions:

We live in a society where many a lot of people are lonely, living as a single person household. Connection is super important. Face‐to‐face communication with other people is a part of mental well‐being. So, if you don't know anybody in a local area … you can go to the park, meet somebody … that's the first person you've spoken to all day, it's a recognition and acknowledgement that you exist as a person. (Male, 76 years, Community).

## 6 Barriers and Facilitators of Green Space Use and Activity Engagement

### 6.1 Neighbourhood and Green Space Characteristics

Participants noted that neighbourhood characteristics influence the use of green spaces. Most of them had access to various green spaces with basic facilities such as playgrounds and footpaths. Participants from rural towns and older suburbs reported living in large properties with greenery and trees. Access to green spaces within a 10–15‐min walk was considered a positive neighbourhood attribute, promoting utilisation. TRC officers emphasised that the region's diverse landscapes offer green spaces from forests to minimally vegetated areas, accommodating varied preferences and experiences. They also highlighted accessibility and connectivity as key considerations in green space policy:

I think it's very accessible from people's homes and their walkability to those open space network. (Officer)

Participants from low‐socioeconomic areas noted limited access to green spaces and public transport. They highlighted that these green spaces lack trees and amenities, which limits the time spent there. While efforts exist to integrate features such as scented plants, guide ropes and braille signage for individuals with visual impairments, a lack of disabled parking spaces was noted as a barrier. Participants recommended enhancing accessibility for diverse disability groups and urged better information dissemination on websites to assist in locating accessible green spaces:

It could also be about whether they can't find information about parks. So, I am thinking of how we have to make the web …. More accessible for people with vision disability (Officer)

### 6.2 Safety

Although participants generally perceived their neighbourhoods to be safe, some noted that safety concerns influenced their use of green spaces. Women and older individuals reported that they avoided visiting late evening or early morning, poorly lit, isolated or overgrown areas. Evidence of overnight camping and alcohol consumption, such as fire remains and scattered bottles, made participants feel insecure in some green spaces, leading them to avoid them:

I would not go wandering by myself in Redwood Park. It is too isolated. (Female, 52 years, Community)

Conversely, participants felt a sense of security when accompanied by family members as well as in the presence of other users or in well‐lit areas:

… when it's still dark … you can still use the park, you stay on the footpath where you feel a bit safer because it's lit. (Female, 41 years, Community)

The presence of individuals experiencing homelessness was frequently mentioned as a safety concern. Participants noted that personal belonging and makeshift shelters used by homeless individuals discouraged them from visiting green spaces. TRC officers also reported confrontations between community garden volunteers and homeless individuals, which led them to feel unsafe. They highlighted the need for caution as some homeless individuals may be dealing with underlying mental health issues:

I go out to Nelly Robinson Park if I want to go to a park but not my place because we have got homeless people there. So, you don't want to put yourself in that situation, so I avoid it. (Female, 69 years, Community)

Media coverage of crime, as well as issues related to cleanliness, vandalism and inadequate maintenance, influenced participants' perceptions of the safety and quality of green spaces, discouraging usage:

There is one area which has graffiti that I don't like walking along because it is dirty… they even tried to get rid of it, but they just leave. It looks worse, so I don't like that area. (Female, 64 years, Community)

### 6.3 Seasonality

Weather conditions, such as extreme temperatures, heavy rain and flooding, were also considered as barriers to green space usage. During breeding seasons, swooping birds, such as Magpies, discouraged use, with participants describing the experience as stressful. Concerns about snakes and paralytic ticks also influenced decisions on when and where to visit:

It was too dangerous; I had eight brown snakes around my house last year. Just coming out of nowhere. So, you need to be careful. (Male, 76 years, Community)

## 7 Discussion

This study explored the perceived role and features of green spaces in influencing mental health and well‐being among adults who regularly used or accessed green space in a regional area in Queensland, Australia. The main themes identified were stress relief and restoration, space for physical activity and social connection, and contextual barriers and facilitators of green space use. These findings highlighted the multifaceted contributions of green spaces to mental health and well‐being, as well as their contextual dependencies in regional settings.

Green space provided an escape, stress reduction and self‐reflection, aligning with Markevych et al.'s [6] restoration domains. These findings support the SRT, suggesting that exposure to nature helps reduce mental fatigue and stress [27], and ART, suggesting that spending time in the natural environment can replenish depleted attention [28]. Participants described natural elements, such as soundscapes, wildlife, trees and landscapes as calming, supporting the Biophilic Hypothesis [29]. This hypothesis argues that humans' innate tendency towards nature reduce stress and enhances mental and spiritual health. These restorative effects emphasise the role of green spaces in renewing adaptive capacities drained by daily demands, thereby improving mental health and well‐being.

This study also revealed that green space fostered physical activity and social connection, reflecting Markevych et al.'s [6] capacity building domain, which posits that natural environments promote health through active lifestyles and social ties. Participants valued green spaces as motivating settings for walking, preferring them over indoor spaces. Dog ownership was the main driver of physical activity, fitness enhancement and mental health support. Simultaneously, green space facilitated social connections by enabling spontaneous interaction and community engagement, fostering a sense of belonging and mitigating loneliness. This suggests that green spaces serve as social infrastructure, contributing to community cohesion and individual well‐being [8]. These findings highlight green spaces as environments that foster physical and social well‐being, contributing to both mental and physical health [3, 6].

Furthermore, our findings showed that accessibility, safety and seasonality influenced green space use, consistent with the sociological model which emphasises the roles of the environment and social determinants in shaping behaviours [20, 21]. Accessibility was a key facilitator, with participants emphasising walkability and proximity as enablers of frequent use. Safety concerns, however, posed substantial barriers, particularly for women and older adults, who avoid isolated or poorly lit areas or vandalised areas, as well as spaces occupied by homeless individuals. These findings indicate that perceptions of social and environmental conditions strongly influence motivations and practises of green space use [8]. Seasonal factors introduced further challenges, including extreme weather and wildlife risks, such as snakes, swooping birds and ticks, deterring the use of green spaces. These environmental factors highlight the need for adaptive management [30]. Collectively, these contextual factors influence the restorative and capacity‐building potential of green spaces, reflecting the interplay between individual perceptions, physical infrastructure and ecological conditions [21].

The restorative benefits of green spaces observed in this study align with those of urban Australian studies [11, 30], affirming the psychological value of green spaces across different settings. Consistent with rural and urban research conducted in Australia [11, 17, 30], our findings demonstrate that green space promotes physical activity, particularly walking and dog walking. Similarly, the role of green space as a community hub for social connection extends prior evidence [4, 6, 11]. The protective effect of green space against loneliness aligns with some Australian studies [9, 31], although a systematic review reported an inconsistent relationship [32]. Similarly, evidence from quantitative urban studies on the mediating role of physical activity and social connections in promoting mental health and well‐being is inconclusive [3], suggesting that the qualitative insights in this study should be interpreted cautiously. Accessibility and safety barriers were consistent with research in rural [33] and urban Australia [34, 35]. Furthermore, barriers related to seasonal factors echoed the findings of a study conducted in Melbourne, Australia [36], which suggested that seasonal changes could affect the frequency of green space visits, length of stay and types of activities. However, further investigation is required to understand the influence of seasonality in regional settings.

Importantly, all participants in this study were members of a community group that regularly engaged in walking and outdoor activities in green spaces. As such, participants were likely to have more favourable attitudes and positive experiences with green spaces than the general population [37]. For example, individuals who regularly use indoor spaces, such as gyms [38], or those with limited familiarity, such as migrants [39], may have different needs, perceptions or barriers to green space use. Thus, characteristics may have shaped the emphasis placed on restorative, physical and social benefits of green spaces and should be considered when interpreting the findings.

Enhancing green space quality through enriched natural features, such as trees, water features and wildlife, as well as improved maintenance and safety measures, could enhance the restorative and capacity‐building benefits of green spaces. Improving accessibility, particularly for underserved groups and areas, adding disability accommodations, enhancing public transport links, and ensuring walkable connections, may encourage regular use. Seasonal challenges, such as extreme weather and wildlife risks, can be mitigated with adaptive measures such as warning signage and provision of shade. Additionally, designing green spaces with off‐leash dog areas and amenities such as benches and shelters could further sustain physical activity and social engagement. Overall, findings of this study, grounded in user experiences and contextual realities, could inform strategies to optimise the health benefits of green space in regional settings.

## 8 Strengths and Limitations

Incorporating perspectives from community residents and council officers enriched insights into the usage and management of green spaces, thus strengthening the validity of the study. The integration of frameworks extended the understanding of the role of green spaces in promoting mental, physical and social well‐being and provided insights into contextual factors, including individual, environmental and social factors that influence green space use. However, this study has some limitations. First, volunteer recruitment may have introduced self‐selection bias. Second, due to limited resources, participants were not recruited from remote and very remote areas. Despite this, we recruited participants from urban and rural localities representing various regional settings. Third, despite efforts to include diverse age groups, the sample consisted mainly of middle‐aged and older adults, predominantly female, who were regular green space users. Given variations in the health effects of green space by gender and age [40], cultural background [41] and attitude towards the natural environment [38], findings of this study may not reflect the perspective of the younger or male population, minority groups such as Aboriginal and Torres Strait peoples, migrants or individuals who primarily use indoor spaces for exercise. Future research should quantify relationships between green space and mental health outcomes across diverse regional settings and include broader demographics to inform green space design.

## 9 Conclusions

This research provides valuable insights into the perceived role and features of green spaces that influence mental health and well‐being among adults in regional Queensland, Australia. Green spaces were found to offer restorative benefits, stress relief and mental rejuvenation. Additionally, these spaces fostered physical activity and social connections, contributing to physical and social well‐being, underscoring the multifaceted contributions of green space to mental health and well‐being in regional Australia and affirming the values of green space across different contexts. This study also revealed the importance of contextual factors influencing greenspace use, including accessibility, safety concerns and seasonal challenges. This highlights the need for contextual‐specific approaches to green space planning and management to maximise the potential health benefits of green spaces.

## Conflicts of Interest

The authors declare no conflicts of interest.

## Data Availability

The data that support the findings of this study are available on request from the corresponding author. The data are not publicly available due to privacy or ethical restrictions.
